# H3K36 trimethylation-mediated biological functions in cancer

**DOI:** 10.1186/s13148-021-01187-2

**Published:** 2021-10-29

**Authors:** Chu Xiao, Tao Fan, He Tian, Yujia Zheng, Zheng Zhou, Shuofeng Li, Chunxiang Li, Jie He

**Affiliations:** 1 Department of Thoracic Surgery, National Cancer Center/National Clinical Research Center for Cancer/Cancer Hospital, Chinese Academy of Medical Sciences and Peking Union Medical College, Beijing, 100021 China; 2 Department of Colorectal Surgery, National Cancer Center/National Clinical Research Center for Cancer/Cancer Hospital, Chinese Academy of Medical Sciences and Peking Union Medical College, Beijing, 100021 China

**Keywords:** Histone modification, H3K36me3, Solid tumor, Epigenetic therapy

## Abstract

Histone modification is an important form of epigenetic regulation. Thereinto, histone methylation is a critical determination of chromatin states, participating in multiple cellular processes. As a conserved histone methylation mark, histone 3 lysine 36 trimethylation (H3K36me3) can mediate multiple transcriptional-related events, such as the regulation of transcriptional activity, transcription elongation, pre-mRNA alternative splicing, and RNA m^6^A methylation. Additionally, H3K36me3 also contributes to DNA damage repair. Given the crucial function of H3K36me3 in genome regulation, the roles of H3K36me3 and its sole methyltransferase SETD2 in pathogenesis, especially malignancies, have been emphasized in many studies, and it is conceivable that disruption of histone methylation regulatory network composed of “writer”, “eraser”, “reader”, and the mutation of H3K36me3 codes have the capacity of powerfully modulating cancer initiation and development. Here we review H3K36me3-mediated biological processes and summarize the latest findings regarding its role in cancers. We highlight the significance of epigenetic combination therapies in cancers.

## Background

Chromatin organized by nucleosomes comprises DNA and associated proteins. A nucleosome includes approximately 146 base pairs of DNA wrapped around by a histone octamer composed of four heterodimers of core histone proteins H2A, H2B, H3, and H4. Histone H1 binds to linker DNA between two nucleosomes.

As for H3, in addition to canonical histones H3.1 and H3.2, which are synthesized only during the S phase termed “replication-coupled histones”, eukaryotic cells also express other histone variants [1]. Unlike canonical histones, histone variant H3.3 is expressed throughout cell cycles, termed “replication-independent histone”. Four to five amino acid differences in peptide sequences between canonical and variants indeed provide distinctive functions for them. The quantity of H3.3 is less than H3.1/H3.2 in most mammalian tissues, accounting for 10–20% in somatic cells [1]. Notably, the deposition of H3.3 preferentially occurs at active gene bodies [2, 3]. Studies have demonstrated that H3.3 mutation is crucial for the initiation of certain cancer types.

In the process of histone post-translational modification (PTM), modificatory labels are deposited and removed by specific enzymes, and these enzymes are figuratively called “writers” and “erasers”. PTM is a fundamental approach for regulating histone functions, occurring at both protruding N-terminal tails and the core globular domains. Modification forms include acetylation, methylation, phosphorylation, ubiquitination, and ADP-ribosylation. Histone methylation mainly occurs at the basic side chains of arginine and lysine and includes mono-, di-, and tri-methylation states [4]. The N-terminal tails of histone H3 carry most PTMs [1]. Different histone methylation marks make distinct effects on transcriptional activation. H3K36, H3K79, and H3K4 methylations generally impart activated effects on gene transcription, but H3K9, H3K27, and H4K20 methylation promote gene silencing [5]. The landscapes of histone marks can reflect the transcriptional state of the cellular genome. For example, during cell differentiation, histone methylation marks would show different distribution patterns in primitive or differentiated cells, and the specific histone mark cluster was called “large organized chromatin lysine domains (LOCKs)” in a study [6]. Specifically, H3K4me1, H3K4me3, and H3K27ac constitute “active LOCK” and they occupy a higher proportion of the genome in primitive cells compared to differentiated cells, thereby acting as one dimension to characterize epigenetics of primitive cells [6]. Apart from the role in regulating transcription, histone methylation marks are also involved in other cellular processes, like bivalent genes/gene poising, transcriptional elongation, DNA damage repair, and mRNA alternative splicing. Considering these intricate functions with complicated regulatory networks of histone methylation for genomic regulation, it is significant to explore their role in malignancies.

H3K36me3 participates in many transcriptional regulatory processes, and those processes include oncogenes and cancer suppressors' expression regulation. Besides, H3K36me3 also mediates DNA damage repair to maintain genomic stability which is vital for keeping a normal cell phenotype. Plenty of studies have shed light on H3K36me3’s role in cancer initiation, progression, and metastasis. Moreover, advances about the role of H3K36 methylation writers, erasers, readers, and H3K36 mutations in cancers have been gradually elucidating the precise mechanisms behind the effect of H3K36me3 in malignancies. In this review, we delineate the available evidence about the association between H3K36me3 and cancers. We propose that H3K36me3 is a promising target for epigenetic therapeutics in cancer.

### Epigenetic regulators

H3K36me3 usually enriches active transcriptional protein-coding genes and increases in a 5′–3′ direction towards the ends of transcriptional regions [7–9]. Functional crosstalk between writers, erasers, and readers shapes the distinct landscapes of H3K36me3 under different cellular contexts.

Histone methyltransferases (HMTases) are responsible for adding methyls to specific lysine or arginine residues on histones, so they are termed “writer”. H3K36 methylation is a highly conserved histone mark and includes three methyl states, mono-, di-, and tri-methylation. At least eight kinds of HMTases have been identified to catalyze H3K36 methylation in higher eukaryotic cells. H3K36 mono- and di-methylation are catalyzed by NSD1, NSD2, NSD3, ASH1L, SETD3, SETMAR, and SMYD2 [7, 10], whereas H3 trimethylation on the lysine 36 is catalyzed specifically by SET domain containing 2 (SETD2), which is also known as HYRB and KMT3A, a vital enzyme of the nuclear receptor SET domain-containing (NSD) family [11]. It is commonly accepted that SETD2 is a non-redundant enzyme found to mediate H3K36 trimethylation in mammalian cells [12]. However, a recent study found that SETD5 has a homology structure with SETD2 within SET and Post-SET domain, showing similar residues position of methylation catalytic domain, which can also catalyze H3K36 trimethylation under in vitro histone methyltransferase assay and cellular context [13].

**Fig.1:**
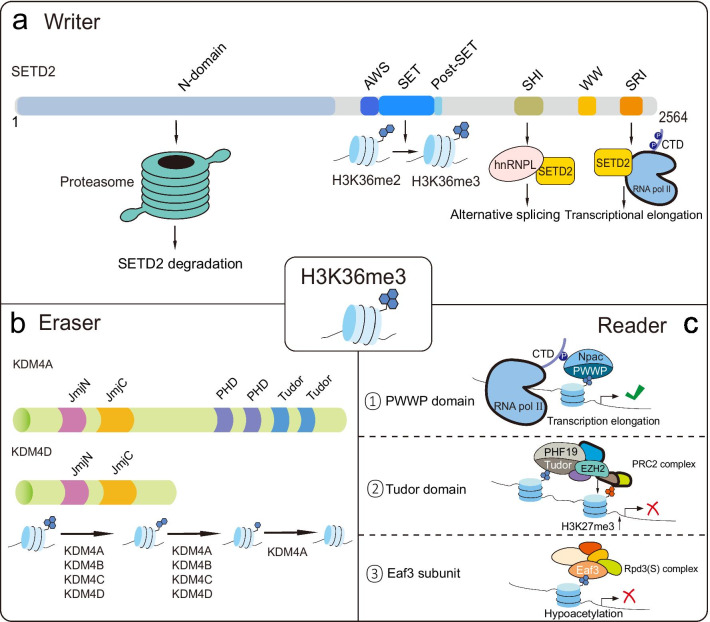
Protein structure and binding sites of H3K36me3-associated epigenetic regulators. **a** SETD2 is the sole histone methyltransferase of H3K36me3. See the text for the function of each domain. **b** The typical protein domain compositions of demethylase family KDM4. **c** Schematic diagram for several critical domains of readers which contribute to binding H3K36me3

SETD2 protein includes three main functional domains (Fig. 1a). First, the methyltransferase activity domains comprise AWS (associated with SET), SET, and PS (post-SET). The SET domain catalyzes H3K36 trimethylation, and missense mutations in this domain will lead to the global loss of H3K36me3. WW (tryptophan-tryptophan), CC (Coiled-Coiled), and SRI (Set2-Rpb1 interacting) are protein binding domains [7]. The SRI domain mediates the interaction between SETD2 and hyperphosphorylated RNA pol II subunit B1 (RPB1) [7, 14]. Recently, Saikat Bhattacharya et al. found that the previously uncharacterized N-terminal region of SETD2, which is only present in mammalian is responsible for the proteasome-mediated degradation of SETD2 [15]. If the segment were removed, the stability of SETD2 would increase significantly and even the formation of insoluble bodies in nuclei. Another study pointed out that the previously uncharacterized SHI domain organizes the interaction between SETD2 and heterogeneous ribonucleoprotein L (hnRNPL) in vitro and in vivo, regulating a common set of genes transcription and alternative splicing events [16]. SETD2 also performs methylation on non-histone substrates such as α-tubulin to affect the genome stability [17, 18].

Like two ends of a balance, demethylases cooperate with methyltransferases to maintain the landscape of H3K36me3 homeostasis. The Jumonji C (JmjC) domain-containing histone demethylases are characterized by the existence of the JmjC domain and are divided into multiple clusters. Each cluster has demethylation activity to specific histone marks [19]. Members of the Jumonji domain 2 (KDM4/JMJD2) cluster can catalyze the demethylation process of H3K36me3/2, including KDM4A/JMJD2A, KDM4B/JMJD2B, KDM4C/JMJD2C, and KDM4D/JMJD2D (Fig. 1b) [19]. KDM4A can remove methyl from all three methylation states of H3K36 [20]. Remarkably, KDM4 family members have been reported to overexpress in various human cancers [21–23], suggesting that H3K36me3 is a tumor suppressor marker.

Readers are referred to as effector proteins that recognize histone modifications and elicit associated biological consequences. The various chromatin-related functions of histone marks at least partially are exerted by these readers. Many protein families can recognize histone PTMs, and these proteins usually possess specific domains to identify different histone modifications. Members from a protein family named Royal family have PWWP, Tudor, chromo, and MBT domains, and they are responsible for interaction with methylated histone tails [24]. Thereinto, Tudor, PWWP, and chromodomain of Eaf3 can bind to H3K36me3 (Fig. 1c) [25]. The PHD finger protein 1 (PHF1) and PHD finger protein 19 (PHF19) from the polycomb-like protein family, are accessory units of the polycomb repressive complex 2 (PRC2, H3K27 methyltransferase) complex, can bind to H3K36me3/2 via the Tudor domain and mediate the recruitment of PRC2 complex to those H3K36me3/2 marked chromatin loci to achieve the shift from active to repressive transcription [26, 27]. The histone demethylase Rpd3 in yeast can identify H3K36m3 and negatively regulate transcription via its subunit Eaf3 [28]. Furthermore, PWWP domain-containing proteins binding to H3K36me3 modulate some critical cellular events. For example, de novo DNA methyltransferase (DNMT) proteins bind to H3K36me3 to regulate DNA methylation, as well as mutS homolog 6 (MSH6), reads H3K36me3 to facilitate DNA mismatch repair. Nucleosome-destabilizing factors (NDF) are a set of proteins that can destabilize nucleosomes and facilitate RNA pol II transcription in chromatin. In humans, glyoxylate reductase 1 homolog (GLYR1/Npac) is a hNDF. Npac deletion can lead to lower enrichment of H3K36me3 marks and globally reduced RNA pol II Ser2, thereby blocking transcriptional elongation [29, 30]. Collectively, the protein–protein interaction between readers and H3K36me3 is vital for maintaining normal chromatin-associated cellular processes. It is possible to restore disordered cellular states by manipulating reader proteins and let them play therapeutic roles.

Apart from writers and erasers, there are additional factors that regulate H3K36 methylation levels at specific sites. Asf1(conserved H3/H4 chaperone) stimulates the SET2 occupancy to the coding regions via binding to H3/H4, thereby promoting the shift from H3K36me2 to H3K36me3 [31]. SPT6, acting as RNA pol II-associated transcription elongation factor and H3 chaperone, can bind to C-terminal domain (CTD) of RNA pol II and recruit Iws1, which recruits SETD2 to the RNA pol II elongation complex to affect histone modification of some active genes like c-Myc, HIV-1, and PABPC1 [32].

### H3K36me3 activates gene expression

Although transcription regulation occurs at many regulatory behavior levels, it can be roughly divided into two forms. One is transcriptional initiation, another is post-transcriptional regulation, including transcriptional elongation and stability [5]. The effect of histone modifications on gene expression is highly variable. Where the marks are placed, which modifications are presented, and which histone modifications coexist all influence the transcriptional regulation process.

**Fig.2:**
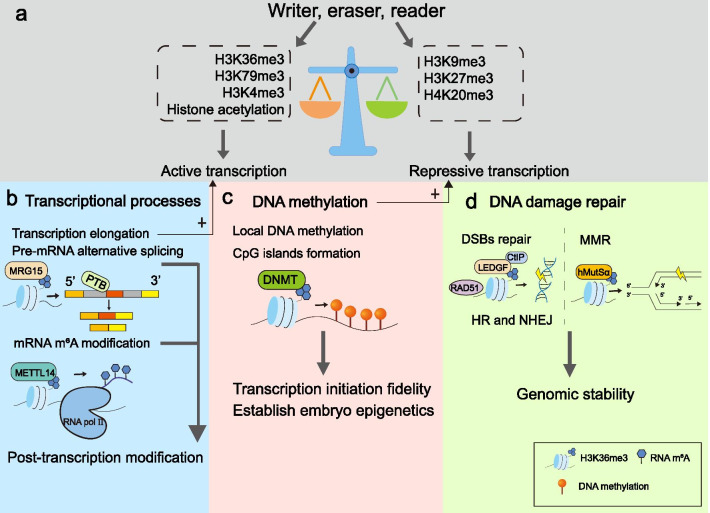
H3K36me3 engages in essential cellular possesses. **a** Active and repressive histone marks maintain gene transcriptional homeostasis. **b** H3K36me3 regulates transcription-related events, including transcription elongation, pre-mRNA alternative splicing, and mRNA m6A modifications. Transcription elongation promotes gene transcription. **c** H3K36me3 affects DNA methylation of local DNA and CpG islands through the interaction with DNMTs. DNA methylation mediated by H3K36me3 represses aberrant transcription. **d** H3K36me3 facilitates MMR and DSBs repair to keep genomic stability

Different histone methylation marks usually have stable-oriented effects on gene transcription (Fig. 2a). Like H3K36me3, H3K4me3, and H3K79me3, they are generally related to active transcription. In contrast, H3K27me3 and H3K9me3 marks dominantly link to silenced chromatin [33]. Intriguingly, the regulative effects will change under different cellular contexts and other concomitant histone modifications. Mechanistically, histone marks serve as binding docks for various transcription-regulated elements to influence gene expression and alter chromatin states [30]. There are plenty of H3K36me3-dependent transcriptional regulation mechanisms waiting to be found and used in pathogenesis.

#### Modifiers regulate transcription through altering genomic H3K36me3 landscape

The writers and erasers of histone marks can regulate gene expression and corresponding cellular functional states by altering the distribution patterns of histone modification. For example, the JmjC proteins belong to the oxygenase superfamily, so under the oxygen stress conditions, the oxygen sensing of chromatin via JmjC enzymes can alter the genomic distribution of H3K4me3 and H3K36me3 which predict transcriptional activation of related genes and then contributes to hypoxia-induced cellular response [34]. Moreover, H3K36me3 mediates the cellular senescence process through the function of methyltransferase NSD2. NSD2 maintains epigenomic states via intrinsic histone methylation ability for H3K36. A study reported that NSD2 upregulates cell cycle-related genes on primary human fibroblasts. NSD2 knockdown would decrease the level of H3K36me3 marks at these gene loci, downregulate gene expression, and induce cellular senescence [35]. Notably, NSD2 is also known as an oncogene for its overexpression in many cancers [36]. In neural stem cells, SETD5 can directly mediate the deposition of H3K36me3 on active gene bodies. Aberrant H3K36me3 distribution owing to SETD5 inactivation can impair the abnormal brain development of mice [13].

Some factors act as the upstream molecules of these modifying enzymes to affect marks occupancy. hnRNPL is a eukaryotic specific subunit of SETD2 complex with sequence preferences, which is required for the activity of SETD2 trimethylated H3K36 in vivo. hnRNPL promotes H3K36me3 to deposit on specific chromatin sites and upregulates corresponding gene expression [37]. In concert with the observation, a recent study reported that hnRNPL binding with seRNA-1 increases the occupancy of H3K36me3 and RNA pol II at myoglobin (Mb) gene locus to enhance myogenic differentiation [38].

Although H3K36me3 is widely perceived as the marker for active gene transcription, emerging evidence indicates that H3K36me3 is associated with heterochromatin formation and gene repression. Chromatin states, classified into euchromatin and heterochromatin, determine the accessibility of promoters for transcriptional factors and the activity of transcription machinery, playing a crucial role in regulating gene expression. SET2 could act as a transcriptional repressor via its SET domain which catalyzes histone lysine 36 trimethylation in *Saccharomyces cerevisiae* [39]. Chantalat et al. found that a widespread H3K36me3 enrichment appeared on the silenced imprint region called Snurf–Snrpn cluster in mouse fetal brains, a highly conserved domain referred to as PWS genomic interval in humans, and the H3K36me3 deposition was independent to RNA Pol II. In this study, H3K36me3 was also detected within constitutive heterochromatin in mouse embryonic stem cells (mESCs) [40]. The underlying mechanisms by which H3K36me3 contributes to the composition of heterochromatin and gene silencing under specific contexts remind to be identified.

#### H3K36me3 is necessary for transcriptional elongation

Transcriptional initiation is usually regulated by the histone marks that are localized at promoter regions. For example, H3K4me3 appears at active gene promoters to promote gene expression while H3K9me3 appears at repressed gene promoters, making the opposite effect on gene expression [33]. Histone marks distributing within gene bodies are likely to regulate transcriptional elongation. In terms of the distribution characteristics of H3K36me3 on chromosomes, that is, H3K36me3 peaks present toward the 3’ end of actively transcriptional regions, it is more convinced that H3K36me3 is involved in transcription elongation, termination, or has a role in transcriptionally linked early mRNA processing [9]. RNA pol II with phosphorylated serine 2 (RNA pol II-2P) is the transcriptionally active elongating form [9]. The SRI domain of SETD2 binds specifically to doubly phosphorylated serine 2 and serine 5 of CTD repeats of RNA pol II, and this interaction is required for K36 methylation on chromatin in yeast and humans [7]. In yeast, SRI domain deletion leads to strong cellular resistance to 6-azauracil (6AU, indictor of transcription elongation defects), and cells with K36 methylation defects due to catalytic inactivation mutations of SET2 or point mutation at K36 were also resistant to 6AU. Taken together, K36 methylation, promoted by SRI domain-dependent association between SETD2 and RNA pol II, is indispensable for the transcription elongation process [14]. However, a study showed no positive correlation between the level of H3K36me3 marks or RNA pol II and transcription elongation rates [41]. The relationship between H3K36me3 and transcriptional elongation needs more elaboration.

#### H3K36me3 and other histone marks collaboratively regulate transcription

H3K36me3 cooperates with other histone modification forms to regulate transcription. As two crucial histone modification forms, histone acetylation and methylation are more referred to as histone marks than other modifications due to their relatively well-defined functions. The two modifications all play essential roles in shaping chromatin states. Histone acetylation marks catalyzed by histone acetylases and deacetylases generally establish open and active chromatin states permissible to transcription [33]. Heterochromatin that represents transcriptional repression is usually characterized by histone hypoacetylation. H3K36me3 can affect gene expression by regulating histone acetylation through the histone modification regulatory network. Rpd3 acts as a transcription repressor targeting promotors in yeast [42], which is contained in two distinct complexes, the small complex is called Rpd3C(S), including Rpd3, Sin3, Ume1, Rco1, and Eaf3. H3K36me3 recruits Rpd3C(S) by its chromodomain subunit Eaf3 and negatively regulates transcription [28, 43], aiming to prevent the initiation of aberrant transcription [44]. Indeed, H3K36me3 can also block spurious intragenic transcripts by interacting with DNMT3B to repress aberrant transcription initiation (see below) [45]. In addition, MST2 is a lysine acetyltransferase, catalyzing H3K14 acetylation and antagonizing heterochromatin. Studies reported that a complex MST2C composed of MST2 includes a protein with a PWWP domain named pdp3, which binds to H3K36me3 and isolates MST2 within the active transcription region in fission yeast [46]. Consistently, the silencing defects at canonical chromatin in set2-deficient cells could be reversed by simultaneous removal of MST2 [46]. This finding supports the idea that histone marks can constrain histone modifiers’ spatial scope of activity to regulate chromatin states.

Given the complexity of histone methylation, the role of the crosstalk between H3K36me3 and other methylation marks in genomic regulation should not be neglected. H3K36me3 can inhibit the activation of PRC2, thereby reducing H3K27me3 occupancy along chromatin and counteracting the H3K27me3-associated transcriptional repression environment in cancer cells [32, 47, 48]. The PRC2 complex comprises EZH2 and the other three core components. EZH2 possesses the SET domain and exhibits methylation activity in the complex [27]. SETD2 can antagonize H3K27me3 effects in prostate cancer by adding methyl to EZH2 to facilitate its degradation [49].

Some histone modifiers target more than one type of histone mark simultaneously. KDM4A can modify all methylation states of H3K9 and H3K36 concurrently. H3K9 methylation is associated with transcriptional repression due to its significant occupancies within heterochromatic regions and the promoters of repressed genes. Although H3K36me3 mainly enriches active genes, the dramatic loss of H3K9me3 and H3K36me3 by KDM4A leads to transcriptional repression [19, 20]. Further, MORF-related gene 15 (MRG15, histone tail-binding protein) can interact with KDM5B, a specific demethylase for H3K4me3/2 in mESCs. H3K36me3 recruits KDM5B to intragenic regions via the interaction between MRG15 and KDM5B and then decreases the level of H3K4me3, thereby restricting cryptic intragenic transcription [50].

The effects of the crosstalk by histone marks on epigenetics are shown in the development of gametes and embryos. SETD2/H3K36me3 plays a crucial role in establishing and safeguarding mouse oocyte epigenome and DNA methylome. A repatterned histone mark distribution form with significantly increased overall genomic distributions of H3K4me3 and H3K27me3 can lead to aberrant gene repression in SETD2-deficient oocytes. These alternations in oocyte epigenome can persist in early embryos and result in embryonic lethality [51]. In addition, NSD1 deficiency-mediated H3K36me2 delation can lead to severe defects in spermatogenesis. [52]. Taken together, these discoveries propose a balance between activated and repressive chromatin modifications, and the activity of modified enzymes is the direct factor in maintaining the poise. A more precise understanding of the regulatory network by multiple histone modification marks will help us clarify how epigenetics modulates cellular functions.

#### DNA methylation mediated by H3K36me3 affects gene activation

Another way H3K36me3 affects transcriptional activity is DNA methylation. DNA methylation is another form of epigenetic regulation, affecting gene expression and transcriptional elongation rates (Fig. 2c) [41]. The DNMT enzymes from the DNMT3 family recognize the H3K36me3 through the PWWP domain [53]. Histone modification regulates the localization of DNA methyltransferases and then modulates DNA methylation levels at promoters, gene bodies, and even genomic-wide range. DNMT3A binds to H3K36me3 and methylates nearby DNA [54]. DNMT3B preferentially targets gene bodies modified by H3K36me3 to participate in DNA methylation or re-methylation [55]. Loss of H3K36me3 from SETD2 inactivation or PWWP domain ablation of DNMT3 would lead to regional loss of DNA methylation [53]. H3K36me3/DNMT is critical for accurate transcription. H3K36me3-mediated recruitment of DNMT3B and subsequent DNA methylation prevents RNA pol II initiation, thereby reinforcing the suppression of spurious transcription and forcing the usage of upstream canonical promoters to ensure transcription initiation fidelity [45]. H3K36me3 regulates not only local DNA methylation but also CpG methylation level. The PWWP domain of DNMT3A can recognize both H3K36me2 and H3K36me3, but it has a higher affinity to H3K36me2. A study reported that NSD1-mediated dimethylation of H3K36 is required to recruit DNMT3A to non-coding euchromatic regions and maintain intergenic CpG methylation levels [56]. The effect of H3K36me3-regulated DNA methylation shows during the initial epigenetic establishment of mice embryos and oocytes [51]. Permissive enrichment in H3K36me3 and reduction in H3K4me3/me2 at imprinting CpG islands assist DNMTs in shaping the right DNA methylome in oocytes [57].

As for the relationship with cancer, aberrant methylation on CpG islands is common in colorectal cancer [58]. The de novo DNA methylation activity is higher at CpG islands marked by H3K36me3 in colorectal tumors, but these methylation targets are similar in normal tissues rather than affected by cancer contexts [58]. Besides, the de novo methylation of genome regions enriched with H3K36me3 was found to keep a high rate after DAC (DNA demethylating agents, 5-aza-2′-deoxycytidine) treatment in normal breast cells [59]. However, in breast cancer, genome regions with high H3K79me2 or H3K4me2/3 levels or aberrantly hypermethylated regions failed to be effectively remethylated after DAC treatment [59]. This finding implies histone marks may serve as a memorize mechanism of DNA methylation. From the perspective of cancer treatment, it makes sense to utilize the conjunctive regulation of DNA methylation and histone modification as a therapeutic target.

### H3K36me3 participates in transcripts processing

#### Alternative splicing

H3K36me3 mediates some pivotal processings of transcripts and post-transcriptional modification. Eukaryotic cells employ alternative splicing to achieve diversification of single gene expression products [60]. H3K36me3 can modulate alternative splicing via several diverse mechanisms. Specifically, H3K36me3 mediates the alternative splicing of PTB-dependent genes through the physical interaction between MRG15 and PTB (polypyrimidine tract binding protein, which is a splicing regulator and can bind to silent elements of exon IIIb of FGFR2 gene). In this adaptor system, MRG15 can specifically bind to H3K36me3 and affect splicing by recruiting PTB to its target exons [61]. Besides MRG15, another protein PC4 and SF2 interacting protein 1 (PSIP1) has also been reported to bind H3K36me3 specifically [62]. PSIP1 encodes two isoforms P52 and P75, and they all harbor the PWWP domain. P52 can specifically bind H3K36me3 and recruit splicing components to chromatin to modulate splicing in mouse embryonic fibroblasts. Instead, P75 does not engage in splicing but interacts with H3K36me3 in DNA damage repair [63].

Notably, the H3K36me3-mediated alternative splicing system is associated with tumor development. FGFR2 can produce two isoforms IIIc and IIIb via alternative splicing, of which only IIIc isoform can be identified by based FGF (bFGF or FGF-2) [64]. IIIc is expressed preferentially in mesenchymal cells and promotes the migration and invasiveness of cells in response to FGF-2, in agreement with the observation that IIIb-to-IIIc switch is related to epithelial-mesenchymal transition and invasive phenotype of cancer cells [64]. SETD2 knockdown would lead to the abundance of the IIIb relative to the IIIc transcripts in NCI H522 and NCI H1299 cells and then attenuate cancer cells migration, invasiveness, and proliferation in the presence of FGF-2 [64]. The lower dense of H3K36me3 on IIIb is a signal for exon inclusion [65].

Another example that supports the potential role of H3K36me3-mediated alternative splicing in cancer is the splicing of EGFR. EGFR-AS1, a lncRNA transcribed from the antisense strand of EGFR, is reported to promote EGFR-A isoform stability and acts as a prognostic in head neck squamous cell carcinoma. The bioinformatic analysis showed that H3K36me3 enriches around the skipped region within EGFR exon 15a and 15b, and the expression of polypyrimidine tract binding protein 1 (PTBP1, belongs to the subfamily of ubiquitously expressed hnRNPs) and its binding site are also enhanced in tumor samples, suggesting that EGFR-AS1 modulates EGFR-A/D isoform expression through alternative splicing conducted by H3K36me3 and PTBP1 [66]. Moreover, H3K36me3 was reported to regulate CDH1 (a tumor suppressor gene in gastric cancer) exon8 splicing in gastric cancer cell lines [67]. Zinc finger MYND-type containing 11 (ZMYND11) which specifically recognizes H3.3 via the PWWP domain also mediates RNA splicing in a SETD2-dependent manner by physical interaction with the RNA spliceosome mechanism. For example, ZMYND11 restrains EFTUD2 (a GTPase which is a component of the spliceosome complex) activation to promote mRNA intron retention (IR), and the binding to H3K36me3 is indispensable for this process [68].

Intriguingly, there is a bidirectional regulation interplay between H3K36me3 and alternative splicing. The H3K36 trimethylation activity of SETD2 depends on pre-mRNA splicing levels. hnRNPL and Aly/Ref1 belong to the complex including SETD2, and they cooperate with RNA pol II to bring SETD2 to the exons, so inhibition spliceosome can reduce the recruitment of SETD2 to chromatin and induce rapid and global redistribution of H3K36me3 levels [16, 37, 69].

#### ***mRNA m***^***6***^***A modification***

N6-methyladenosine (m^6^A) mRNA modification is an important post-transcriptional mechanism of gene regulation. H3K36me3 has been identified as a determinant of m^6^A RNA modification [70]. Strikingly, the 3’UTR deposition patterns of H3K36me3 are similar to m^6^A [71]. METTL14 (an important component of m^6^A methyltransferase complex MTC) identifies and directly binds to H3K36me3 in an RNA pol II-independent manner, facilitating m^6^A MTC to bind adjacent RNA pol II, and then delivering the m^6^A MTC to actively transcribed nascent RNAs for m^6^A deposition during transcriptional elongation (Fig. 2b) [70]. The loss of H3K36me3 significantly reduces transcriptome-wide m^6^A abundance, and the loss of H3K36me3 and m^6^A levels after SETD2 knockdown can be observed on multiple genes like MYC, ACTB, KPNA6, and EEF1A [70]. Hence, regulation of m^6^A modification is a new way for H3K36me3 to control gene expression. However, it is still ambiguous whether there is an association between m^6^A modification and other histone modifications.

### H3K36me3 modulates DNA damage repair

DNA mismatch repair (MMR) ensures replication fidelity and maintains genomic stability via correcting base mismatch and small insertion/deletion, avoiding permanent mutations. Mechanistically, DNA lesions are identified by hMSH2-hMSH6 (hMutSα) and hMSH2-hMSH3 (hMutSβ), and the recruitment of MLH1/PMS2 (MutLα) complex triggers the excision and repair of these mismatches [72]. The subunit of hMutSα hMSH6 contains a PWWP domain by which it binds to H3K36me3 (Fig. 2d), and H3K36me3 promotes MMR by adjusting the distribution of hMutSα [73]. During the cell cycle, SETD2 catalyzes H3K36 trimethylation either in the early S phase or before. Newly formed H3K36me3 specifically recruits hMutSα onto chromatin and then enhances the efficiency of MMR in actively replicated chromatin [73].

In some cancer cell lines, the depletion of SETD2/H3K36me3 can lead to mutant phenotypes. Like SETD2-deficient UOK 143 cells display more microsatellite instabilities [73]. In animal models, a comparison of the mutation distribution of MMR-proficient and MMR-deficient Mlh1^−/−^mice showed that Mlh1^−/−^mice display more distinct microsatellite instabilities, insertion or deletion mutations of repeated sequences, and increased tumor mortality [74]. chromatin Immunoprecipitation (ChIP) and genome-wide DNA sequencing analysis demonstrated that H3K36me3-mediated MMR was mainly employed to protect actively transcribed genes [75]. So the 3′exonic region had fewer MMR-dependent mutations due to the higher density of H3K36me3 compared with 5′exon [74]. Mutations at other amino acid sites on the H3 protein like H3G34 substituted with arginine, valine, or aspartate (H3G34R/V/D) can block the interaction between H3K36me3 and hMutSα due to the steric hindrance effect and prevent the recruitment of hMutSα to chromatin [76]. As a result, cells harboring H3G34R/V/D show an MMR-defective and genomic instability phenotype [76]. Apart from the global MMR facilitation effect, H3K36me3 also protects individual genes. Hume7 and Mcm7, two H3K36me3-enriched active genes during lymphopoiesis, are mutational hotspots in MMR-deficient cells, indicating their intrinsic vulnerability to replication error [74].

DNA double-stranded breaks (DSBs) repair is another important form of DNA damage repair. Unrepair DSBs can result in severe genome instability. DSBs repair includes homologous recombination (HR) and nonhomologous end-joining (NHEJ). HR is a more accurate repair mechanism compared with NHEJ. During the HR process, CtIP and Mre11-Rad50-Nbs1 complex initiate DNA resection to generate ssDNA. Replication Protein A (RPA) binds to ssDNA and leads to RAD51 recombinase (RAD51) nucleofilament formation, ensuring accurate repair [77]. Studies found that H3K36me3 is required for the recruitment of RPA and RAD51 to DSBs sites (Fig. 2d). Abolishment of SETD2 methyltransferase activity, overexpression KDM4A, or introduction of H3.3K36M transgene all exhibit reduction of RPA and RAD51 foci formation and poor HR efficiency, indicating the critical role of H3K36me3 in DSB repair [78]. Moreover, H3K36me3 promotes DSB resection through the interaction with the PWWP domain of Lens epithelium-derived growth factor p75 (LEDGF), a reader protein of H3K36me3 which is responsible for recruiting CtIP to chromatin (Fig. 2d) [63, 78]. LEDGF can bind to lysine acetyltransferase 5 and promote its chromatin location, then stimulates H3K16 acetylation upon DSBs repair inducing H3K36me3 formation in human cells [79].

H3K36me3 is also implicated in the PRDM9 (a histone methyltransferase that catalyzes H3K4me3)-mediated H3K4me3 controlled nonrandom distribution of DSBs during meiotic prophase I of mouse spermatogenic cells [80]. These earlier formed DSBs are inclined to support them to the crossovers (COs) repair process. ChIP analysis demonstrated that the distribution of H3K36me3, H3K27ac, and the focal loss of H3K9me2 showed an overlapping pattern similar to PRDM9-mediated H3K4me3, co-marking more than two thousand DSB hotspots. Thus it is tempting to speculate that these co-marked histone modifications are co-regulated and function coordinately to determine the DSB hotspots [80].

In addition, histone modification can influence specific-site DNA copy number variation. Black et al. found that the catalytic activity of KDM4A would lead to 1q12 copy gain. Moreover, the expression of H3K9me3 and H3K36me3 variants with methionine in place of the lysine was sufficient to promote 1q12h gain [81]. This finding attaches significance to the role of H3K36me3 in cancer from the perspective of DNA replication dysregulation and subsequent genomic instability.

### The role of H3K36me3 in cancers

Epigenetic dysregulation is a hallmark of cancer initiation and progression. Given the crucial role of H3K36me3 in epigenetic regulation, it is expected that H3K36me3 can influence cancer processes through various mechanisms under different cancer contexts. For example, the transcriptional regulatory function of H3K36me3 covers many cancer-related genes in human cell lines. The binding signals of H3K36me3 can predict the expression level of genes. Compared with normal cells, the binding signals of H3K36me3 have the predictive ability for the expression level of oncogenes in cancer cells [82].

Well-balanced epigenetic regulation means the proper modifications formed in the suitable sites at the right time, depending on epigenetic regulators' normal expression and function. Mutations in H3K36me3 writers and erasers are common in tumors and result in a wide range of epigenetic abnormalities, playing stimulative or inhibitory roles in cancer processes (Table 1). Although most epigenetic regulators have been found mutations in cancer genomes sequencing, only a few mutations are effective in cancer. H3K36me3 reduction due to decreased expression or inactivity mutation of SETD2 is observed frequently in various cancers, so SETD2 is generally identified as a tumor suppressor [11]. Demethylases overexpression is common in cancers, leading to some recognized as oncogenes [83–85]. Moreover, mutations of the H3 protein also participate in cancer processes, like H3K36M and H3K36I act as drivers in chondroblastoma [86].

**Table 1:** Overview H3K36me3-associated regulators in different cancers

Cancer subtype	Signaling pathway	Category	Mechanism	Function	Role
Colorectal cancer	Wnt/β-catenin signaling pathway	KDM4C: overexpression	Upregulating MALAT1 expression	Metastasis	Promoter
		SETD2: mutation	Aberrant Dvl2 alternative splicing	Tumorigenesis	Suppressor
Liver cancer	AP-1 pathway	SETD2: mutation	Dysregulated cholesterol homeostasis	Tumorigenesis	Suppressor
			P53 inhibition		
Pancreatic cancer	/	SETD2: mutation	Promoting acinar-to-ductal transition	Transformation	Suppressor
				Progression	
Lung cancer	CXCL1-associated signaling pathway	SETD2: mutation	Aberrant FGFR-2 gene alternative splicing	Tumorigenesis	Promoter
			CXCL1-mediated activation of cell cycle	Proliferation	Suppressor
			P53 inactivation	metastasis	
			Dysregulation of gene expression	Prognosis	
Breast cancer	/	SETD2: mutation	Unstrained expression of oncogenes	Metastasis	Suppressor
		H3 mutation		Prognosis	
Renal cancer	Wnt/β-catenin signaling pathway	SETD2: mutation; loss	Genome reprogramming	Tumorigenesis	Suppressor
			Defective DNA methylation	Prognosis	
			Defective DSBs repair		
			P53 inhibition		
Pediatric cancer	/	H3F3A/H3F3B: mutation	Histone methylation reprogramming	Transformation	Suppressor
		SETD2: mutation; loss	Upregulation of oncogenes	Tumorigenesis	Promoter?
			Impair MMR		
Prostate cancer	AMPK signaling pathway	SETD2: mutation	Altering histone methylation landscape	Progression	Suppressor
Hematological malignant tumor	/	SETD2: mutation	Dysregulation and malignant transformation of hematopoietic stem cells	Tumorigenesis	Suppressor

This table lists the role of H3K36me3-associated epigenetic regulators in different cancer types, including their genetic alternations, related cellular signaling pathways, and which stage of cancer processes their predominantly affect. Pediatric cancer refers to glioblastoma, chondroblastoma, giant cell tumors of bone. The symbol “/” means unknown

S-adenosyl methionine (SAM) is a common substrate for all methylation reactions including histone modification. H3K36me3 is sensitive to SAM pool size during lipopolysaccharide (LPS) -induced inflammation, and LPS stimulation enhances H3K36me3 occupancy at inflammatory genes such as Il1a, Il1b, Il18, and Cxcl12 [87]. Some of these genes are closely related to oncogenesis. Besides, as a one-carbon unit, SAM is an essential metabolite for cancer cell development from the cancer metabolism perspective [88]. More studies on how H3K36me3 participates in cancer metabolism are warranted.

H3K36me3 also cooperates with other proteins to affect tumor cell survival. For example, WEE1 G2 checkpoint kinase (WEE1) inhibition in H3K36me3-deficient tumors can kill cancer cells via synthetic lethality [89]. Mechanistically, loss of ribonucleotide reductase leads to fork stalling and S-phase arrest via deoxyribonucleoside triphosphate exhaustion. RRM2 is a ribonucleotide reductase subunit, and RRM2 deficiency can lead to dNTP depletion and cell apoptosis. RRM2 expression is regulated by two pathways mediated by H3K36me3 and WEE1, respectively. H3K36me3 presenting at RRM2 promoter facilitates its transcription initiation. However, WEE1 inhibition promotes CDK activation-dependent RRM2 degradation via increasing its phosphorylation at T33. So the synergism between WEE1 inhibition and H3K36me3 deletion promotes cancer cell lines like A498, LB996, U2OS, and the xenografts from these cells to death [89]. The role of H3K36me3 in different cancers will be discussed in the following section of the review.

#### Hematological malignant tumor

It has been well-identified that SETD2/H3K36me3 pathway plays a crucial role in the initiation, development, recurrence, and drug resistance in multiple hematological malignancies [90–92]. SETD2 mutations can affect leukemogenic genes expression, impede H3K36me3-mediated DNA damage repair, and induce stress of DNA replication. These aftermaths will result in dysregulated self-renewal and differentiation of hematopoietic stem cells and even cell malignant transformation [93, 94]. More information about the role of SETD2 in hematopoietic malignancies can be found in the review [95]. Except for SETD2, interplays between H3K36me3 marks and other proteins also mediate leukemogenesis [96].

#### Colorectal cancer (CRC)

Studies found the lncRNA-MALAT1 (metastasis-associated lung adenocarcinoma transcript 1) upregulated expression in colorectal cancer and correlated with the tumor grade and metastatic spread. Mechanistically, KDM4C overexpression in colorectal tumor tissues and metastatic samples can lower H3K36me3 and H3K9me3 levels at MALAT1 promotors, thereby upregulating MALAT1 expression and enhancing β-catenin signaling pathway strength, conferring tremendous metastatic potential for CRC cells [97]. In another study, many cancer-associated genes exhibited alternative splicing variations in SETD2 deficient mouse intestines, such as Sirt7, Cdk4, Cdk7, Rab1a, and Lkb1 [98]. SETD2 ablation led to the reduction of H3K36me3 within gene bodies and then increased the production of intron-loss or exon-inclusion transcripts. Notably, the defection of H3K36me3 marks and the suspension of RNA pol II within the intron retentions (IR) area of the dishevelled segment polarity protein 2 (Dvl2) gene resulted in increased Dvl2 pre-mRNA without intron 2, then upregulating Dvl2 expression in CRC cells. Dvl2 plays a pivotal role in promoting the Wnt/β-catenin signaling pathway. Therefore SETD2 loss augments Wnt/β-catenin signaling pathway activity through the dysregulation of Dvl2 alternative splicing [98].

#### Clear cell renal cell carcinoma (ccRCC)

Among all cancers with SETD2 mutations, ccRCC shows the highest mutation rate. 3% ccRCC cases have somatic truncating mutations in SETD2 [83]. According to a study, 11% of Chinese ccRCC patients have SETD2 mutations [99]. Data from 421 TCGA cohorts show that ccRCC cases with SETD2 alternation have higher recurrence frequency and shorter disease-free survival, but the correlation between SETD2 mutation status and over survival is unclear [100].

Multiple cancers have concomitant VHL, PBRM1, and SETD2 mutations, and all these genes map to chromosome 3p. Thereinto, SETD2 is the most commonly mutated gene in ccRCC [83, 101, 102]. The most frequent alternation of chromatin structure in ccRCC involves loss of chromosome 3p [103], so the physical linkage of the three genes determines that the loss of chromosome 3p is the critical driver for tumorigenesis [101]. Mutations in chromatin remodeling genes can impair genome stability and enable cancer cells to maintain carcinogenic phenotypes through genome reprogramming [100]. Epigenetic reprogramming is a central feature of ccRCC, and the reduction of SETD2 can lead to a large-scale transcriptional dysregulation in cancer cells [83], so SETD2 is defined as a driving gene in ccRCC [5]. The different SETD2 mutation frequencies in localized ccRCC and metastatic ccRCC suggest that most SETD2 mutation is subclonal [100]. Mutation in SETD2 is also associated with increased loss of DNA methylation at non-promoter regions, and H3K36me3 reduction can lead to regional loss of DNA methylation [104]. Defect in DNA damage repair is another carcinogenic factor. A recent study revealed that SETD2 is required for HR of DSBs by promoting ATM activation and the recruitment of RAD51 to DSBs. SETD2-depleted cells displayed impaired DNA damage signaling [105]. In addition, TP53-mediated anti-tumor activity is the main obstacle for tumorigenesis in ccRCC due to rare TP53 mutations. However, a study found that loss of SETD2 impedes the P53-mediated checkpoint without the need for P53 mutation, which might disclose why SETD2-deficient cells fail to active P53 even though the persistence of DNA damages [105]. These data identify that SETD2 exerts tumor-suppressive effect via multiple mechanisms in ccRCC. Hanyu Rao et al. proposed that SETD2 is a critical node in the transition from polycystic kidney disease (PKD) to ccRCC. In a c-MYC–driven PKD mouse model, SETD2 inhibited Wnt/β-catenin signaling pathway activity through competing with β-catenin to bind target gene promoters and maintaining the transcriptional levels of the β-catenin destruction complex [106]. Although there are few studies on the association between SETD2/H3K36me3 and cell signaling pathways in cancers, given the above-referred studies of SETD2/H3K36me3 in ccRCC, at least the correlation between SETD2 and Wnt/β-catenin signaling pathway regulatory network is defined to some extent.

#### Pancreatic cancer

The mutation rate of SETD2 is significantly variable among all insertion sites in mice pancreatic carcinoma models [107]. Approximately 3% (n = 775) of pancreatic ductal adenocarcinoma (PDAC) patients carry SETD2 mutations, and nearly all cases with SETD2 mutation have aberrant KRAS, the most common mutation site in PDAC. The cooperation between SETD2 loss and KRAS mutation is considered to promote pancreatic acinar-to-ductal transition and progression [108]. Specifically, SETD2 loss reduces H3K36me3 occupancy at Fbxw7, a well-defined E3-ubiquitin ligase of MYC, leading to decreased Fbxw7 expression and increased MYC protein level. Acinar to ductal metaplasia (ADM) formation is accelerated in this way [108]. Collectively, these studies emphasize again that the fluctuant density of H3K36me3 across the genome can alter the expression of oncogene expression, thereby affecting cell transformation and tumor progression.

#### Lung cancer

H3K36me3 gets involved in lung cancer via multiple pathways. First, H3K36me3 can regulate the alternative splicing of oncogenes, such as FGFR-2 IIIb to IIIc shifts, which promotes lung cancer cell migration and proliferation in response to FGF-2 [64]. Second, SETD2 inhibits CXCL1 gene expression via catalyzing H3K36me3 formation within the promoter of CXCL1 and then indirectly influences related downstream signaling pathways to attenuate the proliferation of lung adenocarcinoma cells and the growth of tumors [109]. In animal models, researchers selectively eliminated SETD2 expression in Kras^LSL−G12D/+^ and Kras^LSL−G12D/+^;p53^flox/flox^ mice. Tumors with H3K36me3 reduction were larger, histopathologically representative like frequent multinucleated giant cells and aberrant mitoses, and had higher proliferation rates, followed by tumors displayed shift from grade 1 adenomas to 2 in Kras^LSL−G12D/+^ mice and grade 3 adenomas to 4 in Kras^LSL−G12D/+^;p53^flox/flox^ mice. The result suggests that SETD2 loss alone is not enough to break through the tumor-suppressor barrier regulated by P53 [110]. Moreover, low SETD2 expression is correlated with reduced overall survival of lung adenocarcinoma (LUAD) patients [110]. The data that 33% (n = 60) LUAD samples with known KRAS or EGFR mutations exhibited low to negative staining of H3K36me3 indicated that SETD2 loss was prevalent in LUAD and had a higher propensity to co-occur with KRAS mutations [110]. Collectively, based on existing studies, SETD2/H3K36m3 acts as a negative regulatory element for lung cancer growth in most cases. More studies are warranted to clarify the antagonistic or synergistic cooperation among SETD2/H3K36me3, P53, and other oncogenes (like KRAS and EGFR) in tumor progression.

#### Breast cancer

Epigenetic dysregulation promotes the process of breast cancer. In primary HR^+^/HER2^−^ subtype, de novo metastatic breast cancer had more SETD2 mutation than recurrent metastatic breast cancer (9.0% [8/89] vs. 2.1% [5/235]) [111]. As a specific reader for H3.3, ZMYND11, known as a candidate tumor suppressor in some cancers [112], can repress oncogenes expression by pausing the transition of RNA pol II to elongation. The deficient binding between ZMYND11 and H3.3K36me3 or ablation of H3K36me3 marks will impair the antitumor effect of ZMYND11, giving rise to the invasive phenotype of MDA-MB 231 breast cancer cells and poorer outcomes of breast cancer patients [113]. However, ZMYND11 promotes malignant transformation when its allele is disrupted due to a chromosome translocation [96]. In a subset of AML patients, the ZMYND11 gene fuses with MBTD1 to form a fusion gene ZM. The introduction of ZM fusion gene into primary hematopoietic stem/progenitor cells is sufficient to cause cell transformation and leukemogenesis in animal models. Mechanistically, the H3.3K36me3 reading ability of ZM protein instructs itself to bind some pro-leukemia genes. Then ZM can facilitate the recruitment of histone acetyltransferase complex NuA4/Tip60 to these genome loci, keeping these oncogenes with higher transcriptional activity upon histone hyperacetylation environment [96]. In addition, SETD2 is one of the most frequent mutated genes in phyllodes tumors of breast, and most cases are inactive mutations along with loss of H3K36me3 [114]. 32% of breast neuroendocrine carcinoma cases (n = 19) with SETD2 loss function mutations show complete loss of H3K36me3, and these mutations can be targeted by histone deacetylase inhibitors [115].

#### Pediatric cancers and stroma

Mutations of histone genes frequently occur in pediatric cancers, such as brain cancer [116] and chondroblastomas/giant cell tumors of bone [86]. The term “oncohistone” refers to those recurrent histone mutations that dominantly drive cancer initiation and development [117]. In humans, H3F3A and H3F3B produce H3.3 proteins. Among various mutations of H3.3, H3.3G34R/V results from H3F3A mutation. A study revealed H3K34 residues in a narrow substrate channel of SETD2, which can block the interaction between SETD2 and H3K36 via steric clashes [76]. The introduction of G34R/V mutant decreases the level of K36me3 at the same or nearby nucleosomes [84, 118], and leads to a differential gene expression signature through interfering H3K36me3 binding pattern and then contributes to gliomagenesis [119]. This H3G34R/V-driven aberrant transcriptional landscape renders many gene upregulations associated with fetal cell decision, especially the expression of MYCN, a potent tumorigenic initiator in glioblastoma [119]. Moreover, defects in H3K36me3-mediated MMR due to H3G34 mutations cause elevated mutation frequency in KNS42 cells [76], which may be another mechanistic explanation of H3G34 mutation in glioma. H3K36me3 also enriches around the promoter of PDK1 gene. The upregulated KDM4A in glioma demethylates H3K36me3 to active PDK1 expression, promoting gliomagenesis via the Akt-mTOR signaling pathway [120].

Different H3.3 mutations promote different cancer types. Heterozygous mutation of the H3 lysine 36-to-methionine (K36M) exists in about 95% of chondroblastomas, and 92% of giant cell tumor cases have lysine 34-to-tryptophan/leucine (G34W/L) mutations [86]. H3K36M in chondroblastoma results in differential expression of cellular development-associated genes, the accumulation of immature chondroblasts, uncontrolled cell proliferation, and incomplete differentiation of mesenchymal progenitor cells [71]. H3K36M/I is a potential inhibition for several methyltransferases like SETD2 and NSD2 by reducing the accessibility of mutant nucleosome to methyltransferases, concomitantly providing new nucleosome substrates for PRC2. This feature of H3K36M/I may explain the observation that H3K36 hypomethylation and H3K27 hypermethylation co-occurrence in cells with H3K36M/I [121]. The intergenic hypermethylation of H3K27 in H3K36M cells alters the level of gene-associated-to-intergenic H3K27me3, resulting in specifically up-regulating some genes repressed by PRC2, such as Wnt6 and Sox6 that mediate the self-renewal of mesenchymal stem cells [121].

In human papillomavirus (HPV)-negative head and neck squamous cell carcinomas, about 13% of cases have epigenome dysregulation. Recurrent K36M and NSD1 mutations disturb the H3K36 methylation landscape with reduced global H3K36me3 and H3K36me2 levels and promote tumorigenesis [122].

Sacral chordoma is a type of rare primary bone tumor. SNP-array combined with NGS analysis demonstrated nonrandom copy number losses across the genome of sacral chordoma patients, frequently involving 3, 9p, 1p, 14, 10, and 13, and two minimum deleted regions which covered SETD2 mapped on chromosome 3p [123]. These findings emphasize that the inactive mutation and deletion of SETD2 may be fundamental genomic aberrations in chordoma. Another study showed that 70% (21/30) of sacrococcygeal conventional chordoma cases had either copy number loss or loss of heterozygosity (LOH) or both of them involving chromosome 3p, supporting the notion mentioned above [124]. Nevertheless, IHC analysis of primary tumor tissues found that patients with lower H3K36me3 levels showed longer relapse-free survival compared with patients with high levels, whereas no significance was found in overall survival [124]. More precise clarifications of which kind of role does SETD2/H3K36me3 plays in sacral chordoma, a carcinomic factor or tumor suppressor, require further evidence.

#### Other cancers

Except for the aforementioned ones, the roles of H3K36me3 and associated epigenetic regulators in other cancer types are pending further studies. We illustrate some new studies in the following sections to improve the functional layout of H3K36me3 in cancers.

Studies showed that SETD2/H3K36me3 expression was reduced in prostate cancer [49]. The physical interaction of SETD2-EZH2 leads to EZH2 K375 methylation and then promotes EZH2 degradation. In this way, SETD2 antagonizes H3K27 methylation to suppress prostate cancer progression [49]. In hepatocellular carcinoma (HCC), RNA-Seq analysis of liver tissues from control and SETD2 KO mice showed that only a fraction of genes changed expression levels upon H3K36me3 reduction [125]. Authors pointed out that impaired H3K36 methylation due to SETD2 ablation mainly interferes with cholesterol homeostasis gene expression in HCC cells, normal liver cells, and SETD2 KO mice, such as ABCA1, ABCG5, and ABCG8. The accumulation of cholesterol puts cells under stress status, which further activates the AP-1 pathway and P53 inhibition, synergistically promoting tumorigenesis [125]. This finding identifies that SETD2/H3K36me3 is indispensable for the normal lipid metabolism of liver cells, which is a determinant of HCC initiation.

The overexpression of KDM4D in gastrointestinal stromal tumor tissues activates the HIF1β gene promoter activity by decreasing the binding of H3K36me3 to the HIF1β gene promoter and induces tumor angiogenesis via the HIF1β/VEGFA signaling pathway [126]. In alveolar rhabdomyosarcoma, increased deposition of H3K36me3 marks at introns and intergenic regions of genes that are downstream of oncogenic transcription factors PAX3-FOXO1 disrupts their transcriptional activity. Using KDM4B inhibitor 17-dimethylaminoethylamino-17-demethoxy-geldanamycin (17-DMAG) to elevate H3K36me3 density can significantly repress tumor growth and improve the survivals of xenograft mouse models [127].

Immunohistochemistry of 135 paraffin-embedded esophageal squamous cell carcinoma (ESCC) samples showed that a lower level of H3K36me3 is correlated with a poorer prognosis of ESCC, and it was speculated that H3K36me3 deficiency may repress tumor suppressor genes expression [128]. Another study also supported that H3K36me3 might be an independent prognostic factor for ESCC patients [129]. It is necessary to clarify whether individual H3K36me3 or combination with other histone methylation marks can act as evaluation indexes for ESCC prognosis and management monitoring. Notably, the prognostic role of H3K36me3 in cancers has been proposed in several cancer types, such as distal bile duct cancer [130].

To date, our understanding of the mechanisms by which H3K36m3 promotes, inhibits, or neutralizes the processes of most cancers is still superficial. More elaborations of the role of H3K36me3 in cancer are urgently needed to further complete the macroscopic impression of epigenetics in cancers.

### Overview of epigenetic therapy in cancer

As an older but developing therapeutic strategy for cancer, the progressive development and application of epigenetic drugs are increasing the anti-cancer strategy options. To date, epigenetic drugs approved by FDA are mainly histone deacetylase inhibitors (HDACis) and DNMT inhibitors (DNMTis). Except for histone acetylation, histone methylation marks are also effective during tumorigenesis and cancer development. For example, mutations in methyltransferase EZH2 [131], SETD2, and DOT1L [132] are common in tumors, and their corresponding histone sites are H3K27, H3K36, and H3K79, separately. Several agents targeting these HMTases are in development. Tazemetostat, which targets EZH2, has been approved by FDA for use in follicular lymphoma. However, its use in solid tumors with hotspot mutations is rare [133]. Inhibitors of DOT1L also have been applied in clinical trials [133].

As for H3K36me3, the known small molecular inhibitors of SETD2 are sinefungin (a nonselective inhibitor for SET-domain containing HMTases) and synthetic nucleoside analogs [134]. A recently published study reported that a series of non-nucleoside inhibitors with novel chemical scaffolds had been developed, which efficiently blocked the methyltransferase activity of STED2. However, only a C13 compound among them showed a decent anti-proliferative effect on two MLL-rearranged AML cell lines and led to reduced H3K36me3 levels with reasonable cellular potency. This chemotype is likely to be an initial hit for drug discovery of AML therapy [135]. In addition, SETD2 can induce synthetic lethality with inhibitors of WEE1 and PI3Kβ-AKT. Cancer cells with SETD2 deficiency or mutations are more sensitive to WEE1 inhibitor Adavosertib, AKT-specific inhibitor, and PI3Kβ-specific inhibitors [136]. Based on the synthetic lethality principle, developing combination therapies may be efficient for selective patients. As for demethylases, various inhibitors of the KDM4 family have been applied in pre-clinical studies [127, 137]. Besides, structural histone protein aberrance from H3F3A and H3F3B mutations are also potential targets for drug development, like histone H3.3 in pediatric tumors [133].

Combining epigenetic therapy and other available tumor therapeutic options in tumor treatment is promising. Once, it was inclined to attribute the functional principle of epigenetic therapy to the correction of aberrant DNA methylation or histone modification that gives rise to malignant phenotypes. Currently, the understandings of epigenetic therapy mechanisms have extended to crossing fields, like tumor immunity, chemosensitivity. Studies have shown that epigenetic agents, like DNMTi, can alter the presence of neoantigen on tumor cells by regulating endogenously methylated sequences, including cancer-testis antigens (CTAs) and human endogenous retroviruses (ERVs) [138]. Then, innate immune response for tumors is triggered, like IFN-γ associated downstream signaling pathways mediated by double-strand RNA (dsRNA), thereby enhancing the patients' response to immunotherapy [139, 140]. Moreover, DNMTi and HDACi are frequently utilized together, as HDACi can reactive genes which are abnormally silenced by promoter DNA methylation [138, 141]. Epigenetic agents also enhance the anti-tumor immunity in ERVs-independent ways. DNA methylation, histone methylation, and histone acetylation have been reported to mediate the functional evolution of tumor-associated cells [142]. Some features of anti-tumor effective cells, like cytokines production and interferon-stimulated genes expression, are suppressed epigenetically during malignancy processes [140, 143]. Meanwhile, the epigenetic reprogramming of non-cancer cells can lead to the pro-cancer phenotype of the tumor microenvironment [144]. Due to the reversibility of epigenetic regulation, DNMTi and histone modification inhibitors can reactivate tumor-effective cells and avoid their exhausted phenotypes, then convert the TME into active anti-tumor states [139, 145, 146]. Preclinical and clinical trials validated that epigenetic drugs augment the efficacy of immunotherapy in solid tumors [141, 147].

Epigenetic therapies are also combined with other system therapies in cancer treatment. The effects of epigenetic agents on multiple cancer-associated mechanisms are the foundation for combination therapies with cytotoxic, targeted, and hormone therapies. For example, the role of HDACis in apoptosis, DNA damage, autophagy induction, senescence induction, hormone signaling regulation, and their related combination therapy paradigms have been elucidated in the review [148]. Mechanistic investigation and preclinical trials suggest that epigenetic agents have chemosensitivity and synergistic efficiency.

Although combination therapies have produced encouraging results, outcomes of epigenetic monotherapy in solid tumor treatments are still unfavorable. The complexity, universality, variability, and vulnerability of epigenetic regulation render specific epigenetic agents primarily limited to specific treatment contexts, which may contribute to the poor therapeutic outcomes in distinct internal environments of patients. Meanwhile, epigenetic alternations generally are not the driver of solid tumors, and unlike active mutations in oncogenes that are easily targeted, most mutations in epigenetic regulators are loss-of-function mutations. So epigenetic treatments are challenging to develop and work inherently [133]. We are still unclear whether epigenome will become a predominant pathway in cancer therapies. A deeper understanding of epigenetic mechanisms is conducive to the development of epigenetic agents.

## Conclusions

Epigenetic regulation mainly includes histone modification and DNA methylation. There are various forms of histone modification, and the regulatory mechanism at least includes “writer”, “eraser”, “reader”, and some additional factors.

Histone modification patterns usually undergo specific alternations during cancer processes, testing the modification profilings has become a novel method for diagnosis and prognostic prediction [128, 129]. For example, circulating nucleosomes harbor the information regarding epigenetics of original cancer cells so that they can be applied as biomarkers for early diagnosis and treatment response monitoring as a part of liquid biopsy [149]. Johan Vad-Nielsen and colleagues verified that H3K36me3 from circulating nucleosomes could quantitatively reflect the transcriptional activity of genes in original cancer cells by cell-free chromatin Immunoprecipitation (cfChIP), which detected histone modifications on circulating nucleosomes [150].

Epigenetic dysregulation is a hallmark for cancer initiation, progression, and metastasis. Hence, chromatin is a very promising therapeutic target in comprehensive tumor therapy. Based on the role mentioned above of epigenetic therapies in tumor immunity and chemosensitivity, we propose that epigenetic combination therapies will be the mainstream application of epigenetic drugs in treating solid tumors. Multiple clinical trials in different solid tumors have achieved encouraging results, like metastatic uveal melanoma (UM) [151], prostate cancer [152], NSCLC [153], and CRC [154], confirming the feasibility of comprehensive therapies. However, formulating criteria of patient populations, therapeutic combination regimens, optimal dosing and schedule, and tolerability all need more information. So more profound and extensive exploration of the biological mechanisms behind combination therapies is warranted to set up theoretical foundations for drug discovery. In addition, available epigenetic drugs are scarce, so developing other epigenetic modification targeted drugs, especially histone methylation, is imperative for increasing combined therapeutic strategies.

Considering that multi-drug combination therapy is prone to eliciting unexpected adverse effects due to complicated drug-drug interaction and interference from biological system elements, the research hotspots have turned to the development of bifunctional epigenetic agents in cancer treatment. Numerous dual-targeted drugs have been created and applied in preclinical trials, such as dual histone deacetylase and demethylase inhibitors [155, 156], dual cyclin-dependent kinase combined with histone deacetylase inhibitors [157], and epigenetic combined cytotoxic agents [158]. It is expected that polypharmacology applied in epigenetic therapy is one of the foundations of drug discovery in the future.

Intriguingly, Bian et al. found that the methionine metabolism of cancer cells disrupts the T cells’ histone mark signature establishment, thereby designing an inhibitor targeted to the methionine transporter of cancer cells for treatment [159]. This finding suggests that we can further explore promising cancer treatment strategies in more fields, like the interplay of cell metabolism and epigenetics, to look for more possibilities.

In this review, we refer to pivotal studies and newly emerging research evidence to summarize the biological functions of H3K36me3, such as gene expression regulation, DNA damage repair, RNA alternative splicing. Furthermore, we analyze the role of H3K36me3 in different types of cancer and delineate known mechanisms behind it. These findings advance our understanding of the role of epigenetics in cancers and have referential values for the development of epigenetic drugs in the future.

## Data Availability

Not applicable.

## Abbreviations

H3K36me3: Histone 3 lysine 36 trimethylation
JmjC: Jumonji C
JMJD2: Jumonji domain 2
PHF1/19: PHD finger protein 1/19
MSH6: MutS homolog 6
NDF: Nucleosome-destabilizing factors
GLYR1: Glyoxylate reductase 1 homolog
mESC: Mouse embryonic stem cell
DDR: DNA damage repair
SETD2: SET domain containing 2
KDM4: Lysine demethylase 4
PTM: Post-translational modification
HMTases: Histone methyltransferases
RNA Pol II: RNA polymerase II
hnRNPL: Heterogeneous ribonucleoprotein L
MMR: Mismatch repair
ZMYND11: Zinc finger MYND-type containing 11
DSB: DNA double-stranded break
DNMT: DNA methyltransferase
HR: Homologous recombination
EGFR: Epidermal growth factor receptor
PSIP1: PC4 and SF2 interacting protein 1
PTBP1: Polypyrimidine tract binding protein 1
RPA: Replication protein A
PRC2: Polycomb repressive complex 2
PTB: Polypyrimidine tract binding protein
ChIP: Chromatin immunoprecipitation
MRG15: MORF-related gene 15
FGFR2: Fibroblast growth factor receptor 2
LPS: Lipopolysaccharide
MALAT1: Metastasis-associated lung adenocarcinoma transcript 1
Dvl2: Dishevelled segment polarity protein 2
EZH2: Enhancer of zeste homolog 2
SAM: S-adenosyl methionine
WEE1: WEE1 G2 checkpoint kinase
PDAC: Pancreatic ductal adenocarcinoma
DOT1L: DOT1 like histone lysine methyltransferase
NSD1: Nuclear receptor binding SET domain protein 1
CRC: Colorectal cancer
ccRCC: Clear cell renal cell carcinoma
LUAD: Lung adenocarcinoma
AML: Acute myeloid leukemia
HCC: Hepatocellular carcinoma
ESCC: Esophageal squamous cell carcinoma
DNMTi: DNA methyltransferase inhibitor
HDACi: Histone deacetylase inhibitor
ERVs: Endogenous retroviruses
